# First experience with FGF-3 (INT-2) amplification in women with epithelial ovarian cancer.

**DOI:** 10.1038/bjc.1993.206

**Published:** 1993-05

**Authors:** A. Rosen, P. Sevelda, M. Klein, K. Dobianer, C. Hruza, K. Czerwenka, H. Hanak, N. Vavra, H. Salzer, S. Leodolter

**Affiliations:** Department of Gynecology and Obstetrics, Hanusch Medical Center, Vienna, Austria.

## Abstract

**Images:**


Br. J. Cancer (1993), 67, 1122–1125
© Macmillan Press Ltd., 1993

### First experience with FGF-3 (INT-2) amplification in women with epithelial ovarian cancer

A. Rosen[1], P. Sevelda[2], M. Klein[1], K. Dobianer[3], C. Hruza[3], K. Czerwenka[2], H. Hanak[4], N. Vavra[2], H. Salzer[2], S. Leodolter[5], M. Medl[5] & J. Spona[2,3]

[1]*Department of Gynecology and Obstetrics, Hanusch Medical Center, Vienna;* [2]*1st Department of Gynecology and Obstetrics, University of Vienna, Vienna;* [3]*Ludwig Boltzmann Institute for Experimental Endocrinology, Department of Cellular Endocrinology, Vienna;* [4]*Department of Pathology, Hanusch Medical Center, Vienna;* [5]*Department of Gynecology and Obstetrics, Lainz Medical Center, Vienna, Austria.*

**Summary** Estimation of FGF-3 oncogene amplification in DNA samples extracted from paraffin embedded sections of 136 ovarian cancer samples was carried out by a quantitative PCR method. The aim of this study was to elucidate a possible association of FGF-3 copy numbers with established prognostic factors such as age, histology, FIGO stage, grading, postoperative residual tumour mass, ascites, hormone receptor content and preoperative CA 125 serum levels. In addition, correlation of FGF-3 amplification with overall survival of the patients was assessed. There was a borderline positive correlation between preoperative CA 125 serum levels and the degree of amplification of the FGF-3 gene ($P = 0.06$). A statistically significant association of FIGO-stage with FGF-3 copy number could be found ($P = 0.008$). No correlation between FGF-3 amplification and overall survival was noted. The data combine to suggest that FGF-3 is an indicator of aggressiveness of ovarian cancer.

The FGF-3 (Fibroblast growth factor 3) gene was found to be located at the second known common integration site for Mouse Mammary Tumor Virus (MMTV) (Peters *et al.*, 1983; Dickson *et al.*, 1984; Peters *et al.*, 1984). The MMTV does not possess a viral oncogene and transforms cells by activation of a cellular protooncogene in the vicinity of the proviral integration site. The FGF-3 gene was thought to be a candidate gene for proviral activation in malignant transformation of the breast. The gene was initially termed INT-2.

Both the genomic sequence of the FGF-3 gene as well as the amino acid sequence of the predicted protein revealed a high homology to the sequences of the acidic and basic fibroblast growth factors (Dickson & Peters, 1987). The human homologue of FGF-3 has been mapped to chromosome 11q13 (Casey *et al.*, 1986; Moore *et al.*, 1986). Although the relevance of FGF-3 to human carcinogenesis had not been demonstrated various observations in mammary tumours had suggested that FGF-3 is a proliferation marker (Adnane *et al.*, 1989; Liderau *et al.*, 1988). It was described that amplification of FGF-3 correlated with steroid receptor levels, recurrence and lymph node status in mammary carcinoma (Liderau *et al.*, 1988; Adnane *et al.*, 1989). In addition, two other genes, PRAD1 and EMS1 (Schuuring *et al.*, 1992), have been described recently which are also located on chromosome 11q13. They were found to be co-amplified with FGF-3, HSTF1 and BCL1 in 40 breast and squamous cell carcinomas as well as nine cell lines. PRAD1 and EMS1 overexpression was found to correlate with amplification (Schuuring *et al.*, 1992). FGF-3 overexpression however was not observed in tumours which exhibit 11q13 amplification. The two newly discovered genes PRAD1 and EMS1 that possibly belong to an amplification unit of about 2,000 kb could contribute to the growth advantage of tumours with 11q13 amplification. The FGF-3 gene which is located within this region could serve as marker for 11q13 amplification. The present experiments were performed to investigate whether FGF-3 amplification could be an indicator for poor prognosis.

Correspondence: J. Spona, 1st Department of Gynecology and Obstetrics, University of Vienna, Spitalgasse 23, E 900, A-1090 Vienna - Austria.
Received 27 August 1992; and in revised form 17 December 1992.

### Patients and methods

One hundred and thirty-six patients operated on for primary ovarian cancer at three different gynecologic departments (Department of Gynecology and Obstetrics/Hanusch Medical Center, 1st Department of Gynecology and Obstetrics/ University of Vienna, Department of Gynecology and Obstetrics/Lainz Medical Center) between 1983 and 1989 were studied. Patients with borderline tumours were excluded from survival statistics, because of their different biologic behaviour.

We compared the amplification of FGF-3 with classical prognostic factors such as FIGO stage, histologic grading, residual tumour, ascites, hormone receptors (estrogen, progesterone) and CA 125 levels prior to and after surgery. The tumour status was evaluated by CT or sonography according to UICC criteria. The tumour stages were assessed by FIGO criteria as of 1985. The histologic grading was according to the criteria established by Day *et al.* (1975), i.e. highly differentiated tumours were classified as grade 1 and undifferentiated tumours were classified as grade 3. Carcinomas with low malignant potency were classified as grade 0 and were defined as borderline tumours (Day *et al.*, 1975). Histologic tumour type was evaluated according to WHO criteria (Serov *et al.*, 1973).

All patients underwent abdominal hysterectomy with bilateral removal of adnexae and omentectomy. Pelvic lymphonodectomy was only performed with a residual tumour mass $<2$ cm. Postoperatively, all patients received a polychemotherapeutic regimen containing cisplatin (six cycles, dosage 75–100 mg $m^{-2}$ cisplatin). A standardised follow-up was performed in an oncologic OPD in 3-monthly intervals during the first 2 years, followed by 6-monthly intervals thereafter.

Tissue samples were frozen in liquid nitrogen immediately after surgery. Preparation of cytosol and nuclei was carried out by homogenising the tissue samples by an Ultra Turrax in 50 mM phosphate buffer at a pH of 7.5. All procedures were carried out at 4°C. The homogenate was centrifuged at 50,000 *g* for 1 h. The supernatant containing the cytosol fraction was used to determine the estrogen receptor (ER) and the progesterone receptor (PgR) concentration. ER and PgR levels were determined by a dextran coated charcoal method by simultaneous incubation with $^{125}$I-estradiol and $^{3}$H-R5020 as ligands as decribed previously (Grill *et al.*, 1984). Data were processed by Scatchard plot analysis (Scatchard, 1949). Cut-off points for ER and PgR were set at

10 fmol mg$^{-1}$ cytosol protein. Tumours with ER and PgR content $<$10 fmol mg$^{-1}$ were classified as hormone receptor negative.

Serum levels of CA 125 were determined by radioimmunoassay with materials obtained from CIS, GIF-sur-Yvette, France. Intra- and interassay coefficients of variations were between 5% and 9%. Blood was sampled 1 day before surgery for preoperative CA 125 serum levels and 4 weeks after surgery for postoperative ones, respectively.

High molecular weight DNA was extracted from frozen tissue sections of about 500 mg of ovarian cancer tissue by standard methods (Sambrook *et al.*, 1989). The $OD_{260}$ was measured in order to estimate the DNA content of the solution. Dot blotting was carried out by fixing 5 μg of DNA to nylon membranes according to standard protocols (Sambrook *et al.*, 1989). The β-actin gene was used as reference gene in dot blot experiments. DNA from frozen placenta tissue served as single copy control.

PCR (polymerase chain reaction) experiments were performed with DNA extracted from paraffin embedded sections of ovarian cancer tissue. Two 10 μm sections were deparaffinised with 1.5 ml isooctane at 70°C for 5 min. After four extraction cycles the samples were dried in a SpeedVac concentrator and incubated with 75 μl lysis buffer (1 mM $CaCl_2$, 0.5% Tween 20, 10 mM Tris HCl, pH 8.0) containing 20 μg proteinase K at 56°C for 4 h. After boiling the samples for 20 min the supernatant was used for PCR runs. Primers PC03 (5'ACACAACTGTGTTCACTAGC3') and KM38 (5'TGGTCTCCTTAAACCTGTCTT3') were used for the β-globin gene as published previously (Saiki *et al.*, 1987). These oligonucleotides yielded a 168 bp PCR product. Primers FGF3a (5'CAGAAGCAGAGCCCGGATAA3') and FGF3b (5'ACGCCAAGATGTCGCCAGGA3') were designed by a computer programme (Rychlik & Rhoads, 1989). These FGF-3 primers resulted in a 130 bp PCR product. All primers were obtained from Biomedica, Vienna, Austria. Three μl of the DNA extraction supernatant were vortexed with 97 μl of reaction mix (0.2 mM dNTP, 0.5 μM of each primer: FGF3a, FGF3b, PC03, KM38, 3 units/100 μl Promega-Taq polymerase, 50 mM KCl, 5 mM $MgCl_2$, 10 mM Tris.HCl pH 8.4, 0.001% gelatine, 0.1% Triton X 100). The PCR samples were incubated in a Thermocycler (Bio-med PCR Processor; start cycle: 94°C; 55°C; 72°C for 2 min each; 30 cycles: 94°C for 30 s; 55°C for 30 s; 72°C for 1 min 30 s; extension time 1 s each cycle and 5 min last step). The PCR products were separated by agarose gel electrophoresis, visualised by ethidium bromide staining and scanned by a densitometer. The oncogene copy number was estimated from the ratio of peak areas using placental DNA as single copy control. The β-globin gene which maps to the same chromosome as the FGF-3 gene was used as a reference in order to account for DNA ploidy in the tumour samples.

### Statistics

For basic data description common statistical parameters such as frequency, mean values and percentages are given. Correlations were quantified by Kendall's tau-c and were tested according to Brown and Benedetti (Brown & Benedetti, 1977). Contingencies in tables of nominal variables were tested by the chi-square test and by Kruskal-Wallis non-parametric analysis. The level of significance was chosen at $P < 0.05$.

The probability of overall survival was assessed according to Kaplan and Meier and was calculated by the Mantel-Cox log-rank test (Kaplan & Meier, 1958; Mantel, 1966).

### Results

The results of the PCR method correlated with dot blot assays showing a correlation coefficient of 0.80, a slope of 0.74 and an intercept of 0.31 in linear regression analysis (data not shown). These experiments were performed to demonstrate the validity and reproducibility of the newly developed PCR procedure. In 136 patients with epithelial ovarian cancer the FGF-3 copy numbers were determined by this quantitative PCR. The median age of the patients was 61 years with a range between 27 and 88 years. The women were observed over a period of 12 to 79 months with a median follow-up period of 28 months. All other patient characters such as FIGO-stage, histologic grading, histologic type, status of hormone receptors, ascites and residual tumour mass are shown in Table I.

Tumour samples with less than 1.5 copies were defined as not amplified and classified as single copy tumours, whereas tumour samples with more than 1.5 copies were defined as amplified (Figure 1). One hundred and nine out of 136 (80%) tumour samples showed single copy FGF-3 oncogene, whereas 27/136 (20%) ovarian tumours had an amplified FGF-3 gene with copy numbers between 1.5 and 3.5. Tables I and II show these data with regard to amplification of the FGF-3 oncogene.

Preoperative CA 125 levels were above the established cut-off of 35 IU ml$^{-1}$ in 112/136 (82.6%) patients and a borderline significance ($P = 0.06$) for patients with amplified FGF-3 gene as compared to patients with single copy gene was noted (Table II). In 74/136 (54.8%) subjects hormone

**Figure 1** FGF-3 PCR products on an agarose gel. Lane 1: Placenta; lanes 2, 6, 7: samples with single copy FGF-3 gene; lanes 3, 4, 5, 8: samples with amplified FGF-3 gene; lane M: molecular weight marker (pBR322/HaeIII digested).

**Table I** Clinicopathological parameters and association with FGF-3 copy numbers

| | *FGF-3 single copy* | | *FGF-3 amplified* | | *CHI-square* |
|---|---|---|---|---|---|
| *FIGO-stage* | | | | | |
| I | 30 | 27.52% | 0 | | |
| II | 4 | 3.67% | 3 | 11.11% | |
| III | 65 | 59.63% | 19 | 70.37% | $P = 0.008$ |
| IV | 10 | 9.17% | 5 | 18.52% | |
| *Histol. grade* | | | | | |
| GO | 8 | 7.41% | 0 | | |
| GI | 21 | 19.44% | 3 | 11.11% | $P = 0.17$ |
| GII + GIII | 79 | 73.15% | 24 | 88.89% | n.s. |
| *Histology* | | | | | |
| serous | 65 | 59.63% | 17 | 62.96% | |
| mucinous | 14 | 12.84% | 1 | 3.70% | |
| endometr. | 5 | 4.59% | 2 | 7.41% | $P = 0.671$ |
| undiff. | 13 | 11.93% | 3 | 11.11% | n.s. |
| clearcell | 6 | 5.50% | 1 | 3.70% | |
| other | 6 | 5.50% | 3 | 11.11% | |
| *Ascites* | | | | | |
| yes | 54 | 50.00% | 12 | 44.44% | $P = 0.60$ |
| no | 54 | 50.00% | 15 | 55.56% | n.s. |
| *Residual tumour mass* | | | | | |
| none | 44 | 40.37% | 6 | 22.22% | |
| $<$2 cm | 23 | 21.10% | 9 | 33.33% | $P = 0.173$ |
| $>$2 cm | 42 | 38.53% | 12 | 44.44% | n.s. |
| *Steroid receptor levels* | | | | | |
| pos. | 40 | 36.70% | 11 | 42.30% | |
| neg. | 18 | 16.50% | 5 | 19.20% | $P = 0.74$ |
| none | 51 | 46.80% | 10 | 38.50% | n.s. |

**Table II** Association of steroid receptor levels and preoperative CA 125 serum levels with FGF-3 copy numbers

| | | *FGF-3 single copy* | *FGF-3 amplified* | *Kruskal-Wallis CHI-square* |
|---|---|---|---|---|
| *Age* | median | 60.1 a | 57.8 a | $P = 0.17$ |
| ($n = 136$) | range | (27–88) | (39–80) | n.s. |
| *Pg-receptor* | median | 0 fmol ml$^{-1}$ | 8 fmol ml$^{-1}$ | $P = 0.28$ |
| ($n = 74$) | range | (0–467) | (0–221) | n.s. |
| *E-receptor* | median | 12.5 fmol ml$^{-1}$ | 11 fmol ml$^{-1}$ | $P = 0.81$ |
| ($n = 75$) | range | (0–196) | (0–166) | n.s. |
| *CA 125* | median | 193 IE ml$^{-1}$ | 382 IE ml$^{-1}$ | $P = 0.06$ |
| ($n = 134$) | range | (3.5–> 10.000) | (8–> 10.000) | |

**Figure 2** Survival probability in epithelial ovarian cancer by Kaplan-Meier analysis. Single copy FGF-3 oncogene *vs* amplified FGF-3 oncogene.

receptor levels could be estimated (Table II). In the single copy group there were 40 women (36.7%) with positive hormone receptor findings *vs* 11 (42.3%) women in the amplified group. Eighteen (16.5%) patients with negative hormone receptor findings in the single copy group were found compared to 5 (19.2%) patients with negative findings in the amplified group. No significant difference was detected.

Thirty out of 136 patients were FIGO stage I and all of them showed single copy FGF-3 oncogene. This correlation was highly significant ($P = 0.008$). Concerning the histologic grade, the FGF-3 gene turned out to be not amplified in all patients with G0 turnouts. Fifty out of 136 patients did not have any residual tumour. In 44 of these 50 cases a single copy FGF-3 gene was found and 6/50 cases contained an amplified FGF-3 gene. No significant association of age, estrogen receptor and progesterone receptor status with FGF-3 amplification was observed.

No significant association of overall survival with FGF-3 amplification was found (Figure 2).

### Discussion

This study provides the first evidence of the amplification of the FGF-3 oncogene in patients with ovarian cancer. In breast cancer the FGF-3 oncogene seems to be one of the three most frequently amplified oncogenes (Adnane *et al.*, 1989). Amplification frequencies have been reported to range between 4% and 23% of cases of mammary carcinoma (Varley *et al.*, 1988; Zhou *et al.*, 1989). In the present study on ovarian cancer the FGF-3 gene was found to be amplified in a similar percentage of cases, since 20% of the ovarian cancer samples investigated were found to have an amplified FGF-3 oncogene.

Prognostically favourable groups could benefit from the presence of a single copy of the FGF-3 gene, because all FIGO stage I, all GO and 88% of the cases with no residual tumour had a single copy number. Except for the association of FIGO stage with FGF-3 copy number ($P = 0.008$) no other correlations between FGF-3 amplification and clinicopathological indices could be found. In addition, no influence of FGF-3 copy number on overall survival was noted. This could at least partly be explained by the small patient numbers in this investigation.

CA 125 is a tumour antigen, which was reported by Bast *et al.* in 1983, and its validity as a prognostic factor was subsequently confirmed (Sevelda *et al.*, 1987; Sevelda *et al.*, 1989; Rosen *et al.*, 1990; Sevelda *et al.*, 1991). Significant correlation between CA 125 and the classical prognostic factors such as residual tumour mass, histological grading, ascites and FIGO stage was reported recently (Makar *et al.*, 1992). These parameters characterise the biologic properties of a tumour. No correlation ($P = 0.06$) between preoperative CA 125 serum levels and FGF-3 amplification could be observed in the present investigation. The borderline association between CA 125 and FGF-3 amplification as well as the significant correlation between FIGO stage and oncogene amplification combine to suggest that FGF-3 could contribute to aggressiveness and tumour proliferation. Larger numbers of patients should however be investigated to support this notion. In addition, levels of FGF-3 expression in ovarian tumours with and without 11q13 amplification should be studied in order to shed light on the possible role of FGF-3 as a proliferation marker.

Part of this work was supported by 'Fond zur Förderung der wissenschaftlichen Forschung Projekt No P 8509 MED'. The support of K. Dobianer by this grant is also gratefully acknowledged.

### References

ADNANE, J., GAUDRAY, P., SIMON, M.P., SIMONY-LAFONTAINE, J., JEANTEUR, P. & THEILLET, C. (1989). Proto-oncogene amplification and human breast tumor phenotype. *Oncogene*, **4**, 1389-1395.

BAST, R.C., KLUG, T.L., JOHN, E. ST., JENISON, E., NILOFF, J., LAZARUS, H., BERKOWITZ, R., LEAVITT, T., GRIFFITHS, C., PARKER, L., ZURAWSKI, V.R. & KNAPP, R.C. (1983). A radioimmunoassay using a monoclonal antibody to monitor the course of epithelial ovarian cancer. *N. Engl. J. Med.*, **309**, 883-887.

BROWN, M.B. & BENEDETTI, J.K. (1977). Sampling behavior of tests for correlation in two-way contingency tables. *J. Am. Stat. Assoc.*, **72**, 309-315.

CASEY, C., SMITH, R., MCGILLIVRAY, D., PETERS, G. & DICKSON, C. (1986). Characterisation and chromosome assignment of the human homolog of INT-2, a potential proto-oncogene. *Mol. Cell. Biol.*, 502-510.

DAY, T.G., GALLAGER, H.S. & RUTLEDGE, F.N. (1975). Epithelial carcinoma of ovary: the prognostic importance of histologic grade. *Natl Cancer Inst. Monogr.*, **42**, 15-21.

DICKSON, C. & PETERS, G. (1987). Potential oncogene product related to growth factors. *Nature*, **326**, 833.

DICKSON, C., SMITH, R., BROOKS, S. & PETERS, G. (1984). Tumorgenesis by mouse mammary tumor virus: proviral activation of a cellular gene in the common integration region INT-2. *Cell*, **343**, 529-536.

GRILL, H.J., MANZ, B., BELOVSKY, O. & POLLOW, K. (1984). Double ligand assay for estrogen and progesterone receptor. *Oncology*, **41**, 25-29.

KAPLAN, E.L. & MEIER, P. (1958). Non-parametric estimation from incomplete observations. *J. Am. Stat. Assoc.*, **53**, 457-481.

LIDEREAU, R., CALLAHAN, R., DICKSON, C., PETERS, G., ESCOT, C. & ALI, I.U. (1988). Amplification of the INT-2 gene in primary human breast tumors. *Oncogene Res.*, **2**, 285-291.

MAKAR, A.PH., KRISTENSEN, G.B., KÆRN, J., BØRMER, O.P., ABELER, V.M. & TROPÉ, C.G. (1992). Prognostic value of pre- and postoperative serum CA 125 levels in ovarian cancer: new aspects and multivariate analysis. *Obstet. Gynecol.*, **79**, 1002-1010.

MANTEL, N. (1966). Evaluation of survival data and two new rank order statistics arising in its consideration. *Cancer Chem. Rep.*, **50**, 163-170.

MOORE, R., CASEY, G., BROOKS, S., DIXON, M., PETERS, G. & DICKSON, C. (1986). Sequence, topography and protein coding potential of mouse INT-2: a putative oncogene activated by mouse mammary tumor virus. *EMBO J.*, **5**, 919-924.

PETERS, G., BROOKS, S., SMITH, R. & DICKSON, C. (1983). Tumorigenesis by mouse mammary tumor virus: evidence for a common region for provirus integration in mammary tumors. *Cell*, **33**, 369-377.

PETERS, G., KOZAK, C. & DICKSON, C. (1984). Mouse mammary tumor virus integration regions INT-1 and INT-2 map on different mouse chromosomes. *Mol. Cell. Biol.*, **4**, 375-378.

ROSEN, A., SEVELDA, P., KLEIN, M., SPONA, J. & BECK, A. (1990). A CA 125 score as a prognostic index in patients with ovarian cancer. *Arch. Gynecol. Obstet.*, **247**, 125-129.

RYCHLIK, W. & RHOADS, R.E. (1989). A computer program for choosing optimal oligonucleotides for filter hybridization, sequencing and *in vitro* amplification of DNA. *Nucleic Acids Res.*, **17**, 8543-8551.

SAIKI, R.K., GELFAND, D.H., STOFFEL, S., SCHARF, S.J., HIGUCHI, R., HORN, G.T., MULLIS, K. & ERLICH, H.A. (1987). Primer-directed enzymatic amplification of DNA with a thermostable DNA polymerase. *Science*, **239**, 487-491.

SAMBROOK, J., FRITSCH, E.F. & MANIATIS, T. (1989). *Molecular Cloning – A Laboratory Manual (2nd Ed)*. Cold Spring Harbor Laboratory Press: USA.

SCATCHARD, G. (1949). Interaction of small molecules. *Ann. N.Y. Acad. Sci.*, **51**, 660-665.

SCHUURING, E., VERHOEVEN, E., MOOI, W.J. & MICHAELIDES, R.J.A.M. (1992). Identification and cloning of two overexpressed genes, U21B31/PRAD1 and EMS1, within the amplified chromosome 11q13 region in human carcinomas. *Oncogene*, **7**, 355-361.

SEROV, S.F., SCULLY, R.F. & SARABIN, L.H. (1973). Histological typing of ovarian tumors. In *International Histological Classification of Tumors*, pp. 17. World Health Organisation: Geneva, Switzerland.

SEVELDA, P., SALZER, H., DITTRICH, CH., PATEISKY, N. & SPONA, J. (1987). The diagnostic validity of the tumormarker Ca 125 in ovarian cancer patients. *Tumor Diagnostik Ther.*, **8**, 115-120.

SEVELDA, P., SCHEMPER, M. & SPONA, J. (1989). Ca 12-5 as an independant prognostic factor for survival in patients with epithelial ovarian cancer. *Am. J. Obstet. Gynecol.*, **161**, 1213-1216.

SEVELDA, P., ROSEN, A., DENISON, U., BARRADA, M., SPONA, J. & SALZER, H. (1991). Is CA-125 monitoring useful in patients with epithelial ovarian carcinoma and preoperative negative CA 12-5 serum levels? *Gynecol. Oncol.*, **43**, 154-158.

VARLEY, J.M., WALKER, R.A., CASEY, G. & BRAMMAR, W.J. (1988). A common alteration to the int-2 proto-oncogene in DNA from primary breast carcinomas. *Oncogene*, **3**, 87-91.

ZHOU, D.J., AHUJA, H. & CLINE, M.J. (1989). Proto-oncogene abnormalities in human breast cancer: c-erbB-2 amplification does not correlate with recurrence of disease. *Oncogene*, **4**, 105-108.


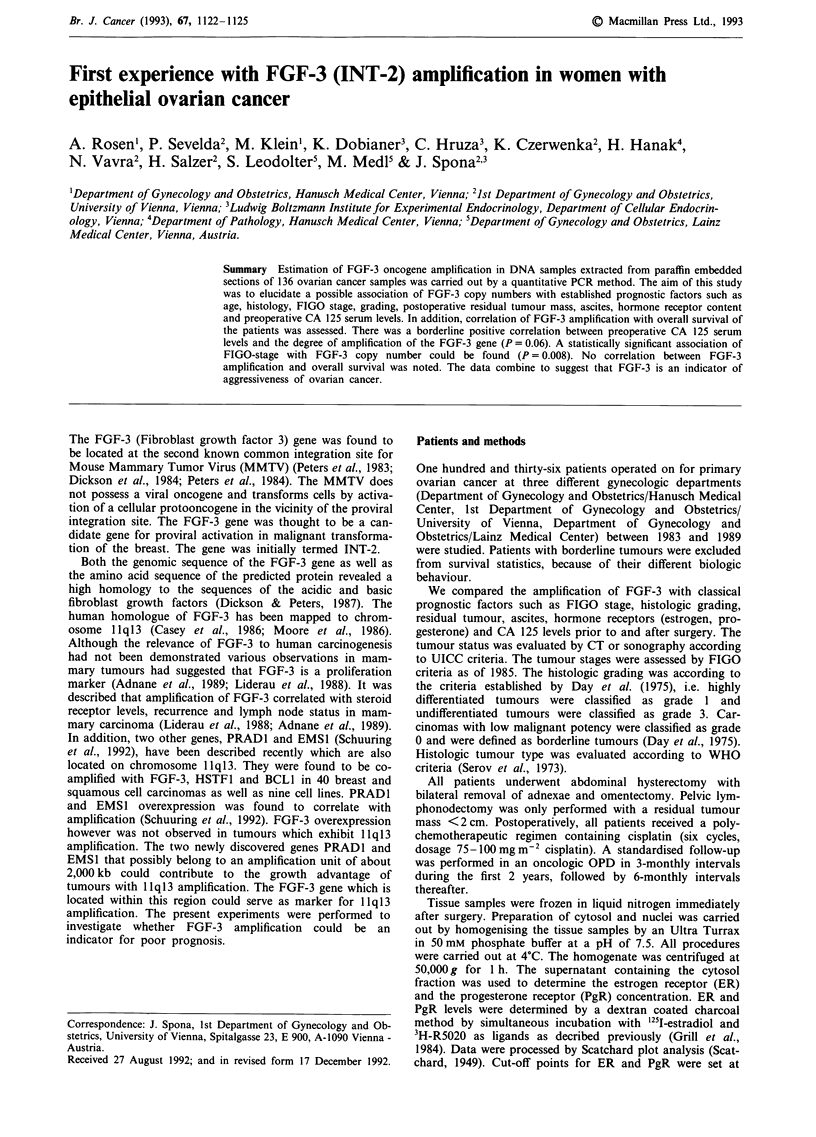

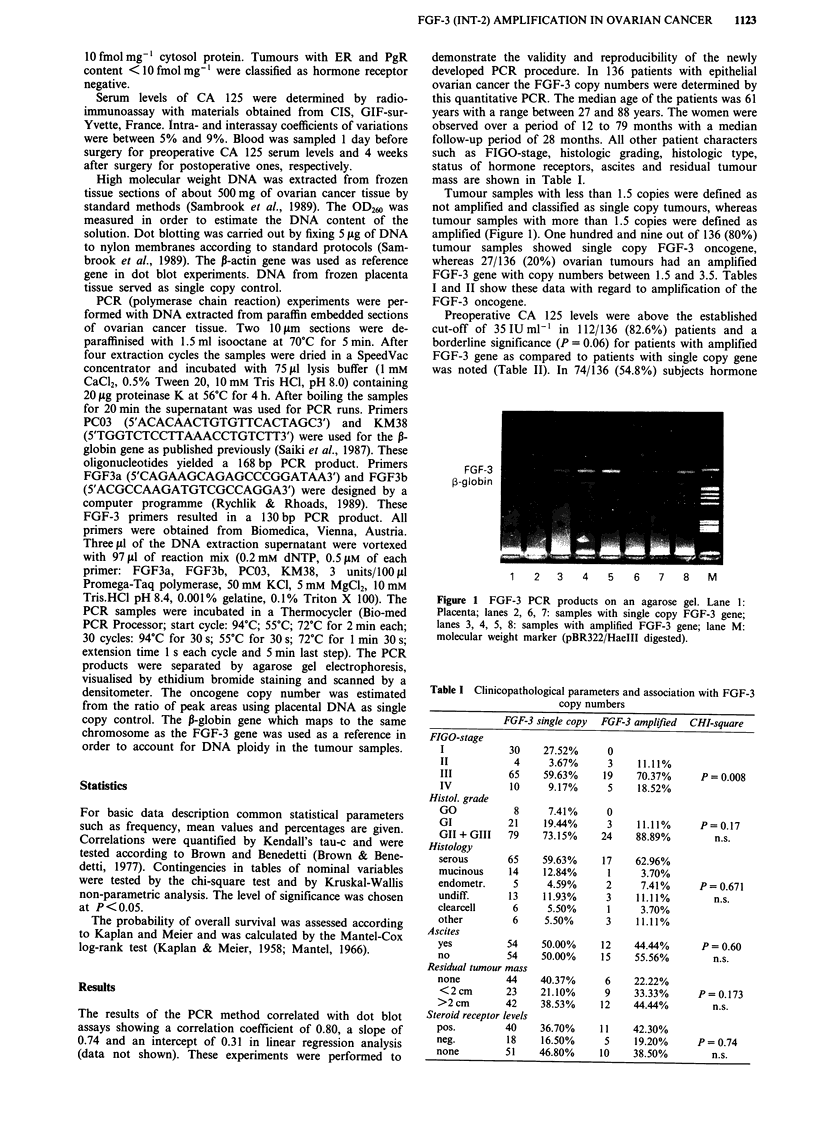

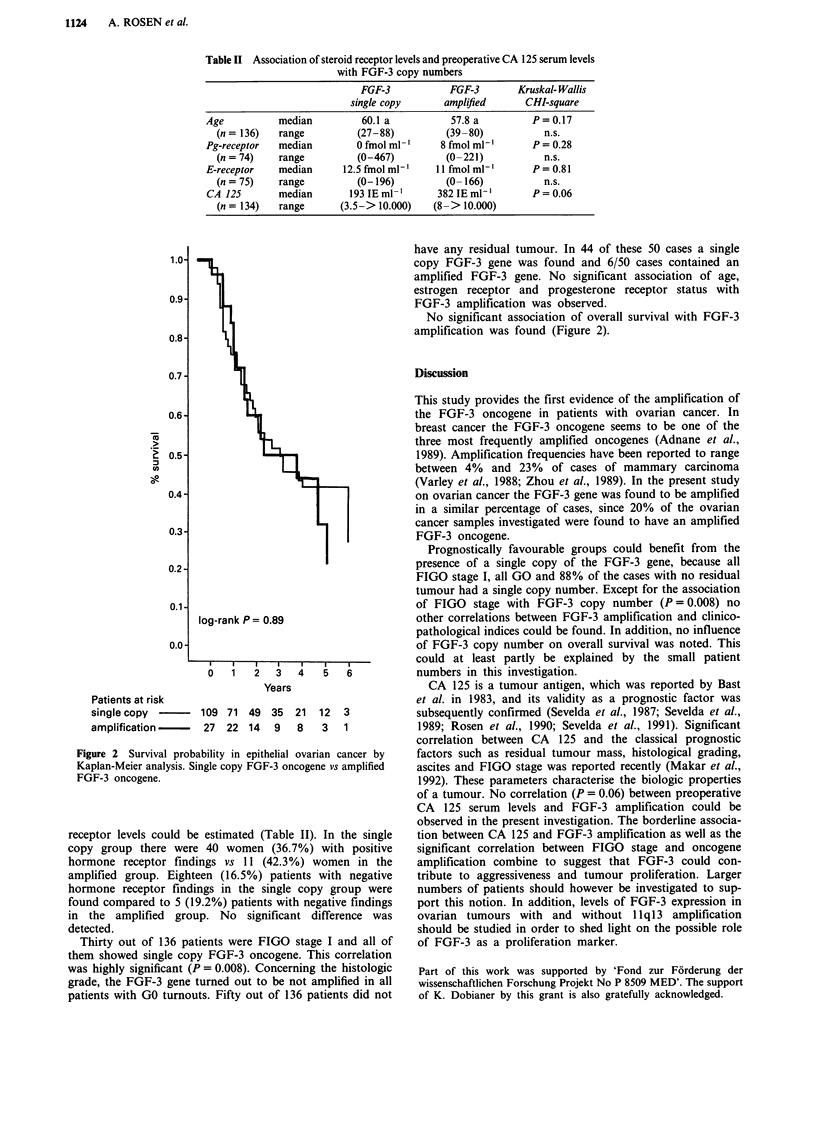

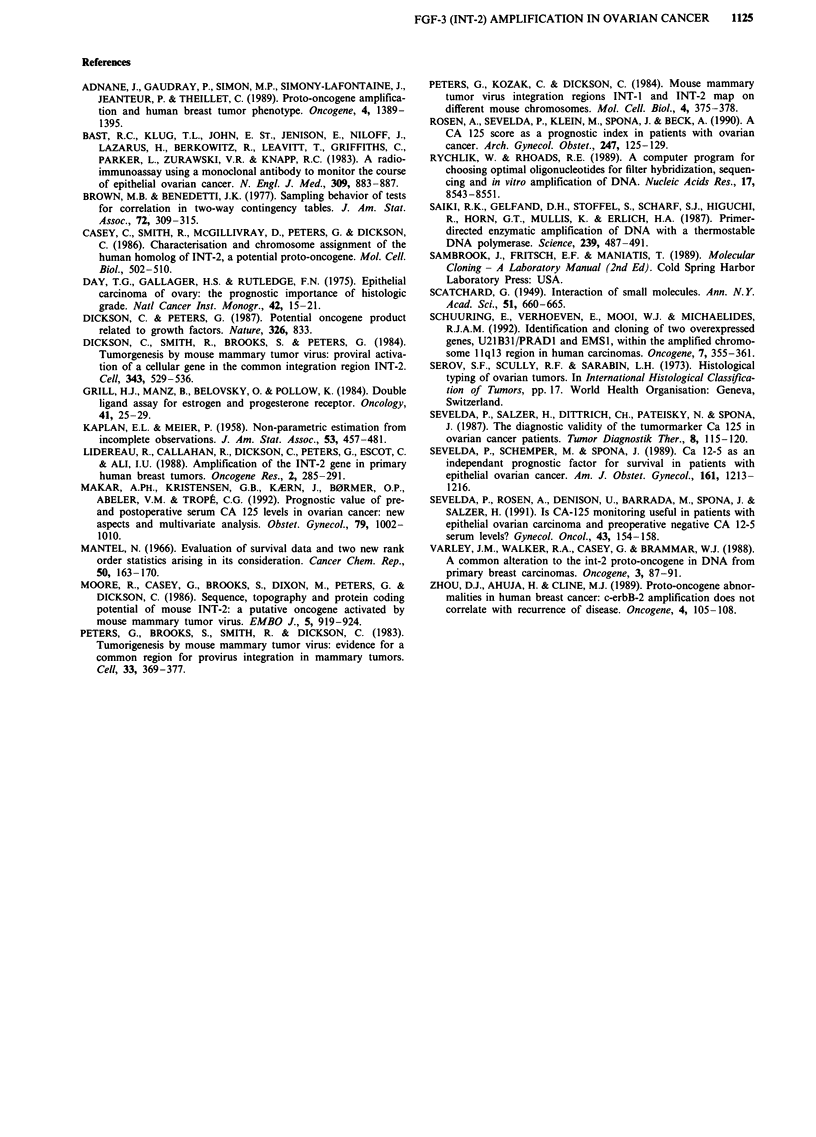


## References

[OCR_00438] Adnane J., Gaudray P., Simon M. P., Simony-Lafontaine J., Jeanteur P., Theillet C. (1989). Proto-oncogene amplification and human breast tumor phenotype.. Oncogene.

[OCR_00444] Bast R. C., Klug T. L., St John E., Jenison E., Niloff J. M., Lazarus H., Berkowitz R. S., Leavitt T., Griffiths C. T., Parker L. (1983). A radioimmunoassay using a monoclonal antibody to monitor the course of epithelial ovarian cancer.. N Engl J Med.

[OCR_00456] Casey G., Smith R., McGillivray D., Peters G., Dickson C. (1986). Characterization and chromosome assignment of the human homolog of int-2, a potential proto-oncogene.. Mol Cell Biol.

[OCR_00462] Day T. G., Gallager H. S., Rutledge F. N. (1975). Epithelial carcinoma of the ovary:prognostic importance of histologic grade.. Natl Cancer Inst Monogr.

[OCR_00467] Dickson C., Peters G. Potential oncogene product related to growth factors.. Nature.

[OCR_00471] Dickson C., Smith R., Brookes S., Peters G. (1984). Tumorigenesis by mouse mammary tumor virus: proviral activation of a cellular gene in the common integration region int-2.. Cell.

[OCR_00574] Eisner J. M., Casey B. J. (1988). Malpractice, informed consent, and the use of low osmolality contrast media.. Conn Med.

[OCR_00477] Grill H. J., Manz B., Belovsky O., Pollow K. (1984). Criteria for the establishment of a double-labeling assay for simultaneous determination of estrogen and progesterone receptors.. Oncology.

[OCR_00486] Lidereau R., Callahan R., Dickson C., Peters G., Escot C., Ali I. U. (1988). Amplification of the int-2 gene in primary human breast tumors.. Oncogene Res.

[OCR_00493] Makar A. P., Kristensen G. B., Kaern J., Børmer O. P., Abeler V. M., Tropé C. G. (1992). Prognostic value of pre- and postoperative serum CA 125 levels in ovarian cancer: new aspects and multivariate analysis.. Obstet Gynecol.

[OCR_00498] Mantel N. (1966). Evaluation of survival data and two new rank order statistics arising in its consideration.. Cancer Chemother Rep.

[OCR_00503] Moore R., Casey G., Brookes S., Dixon M., Peters G., Dickson C. (1986). Sequence, topography and protein coding potential of mouse int-2: a putative oncogene activated by mouse mammary tumour virus.. EMBO J.

[OCR_00509] Peters G., Brookes S., Smith R., Dickson C. (1983). Tumorigenesis by mouse mammary tumor virus: evidence for a common region for provirus integration in mammary tumors.. Cell.

[OCR_00515] Peters G., Kozak C., Dickson C. (1984). Mouse mammary tumor virus integration regions int-1 and int-2 map on different mouse chromosomes.. Mol Cell Biol.

[OCR_00520] Rosen A., Sevelda P., Klein M., Spona J., Beck A. (1990). A CA125 score as a prognostic index in patients with ovarian cancer.. Arch Gynecol Obstet.

[OCR_00525] Rychlik W., Rhoads R. E. (1989). A computer program for choosing optimal oligonucleotides for filter hybridization, sequencing and in vitro amplification of DNA.. Nucleic Acids Res.

[OCR_00531] Saiki R. K., Gelfand D. H., Stoffel S., Scharf S. J., Higuchi R., Horn G. T., Mullis K. B., Erlich H. A. (1988). Primer-directed enzymatic amplification of DNA with a thermostable DNA polymerase.. Science.

[OCR_00546] Schuuring E., Verhoeven E., Mooi W. J., Michalides R. J. (1992). Identification and cloning of two overexpressed genes, U21B31/PRAD1 and EMS1, within the amplified chromosome 11q13 region in human carcinomas.. Oncogene.

[OCR_00568] Sevelda P., Rosen A., Denison U., Barrada M., Spona J., Salzer H. (1991). Is CA-125 monitoring useful in patients with epithelial ovarian carcinoma and preoperative negative CA-125 serum levels?. Gynecol Oncol.

[OCR_00562] Sevelda P., Schemper M., Spona J. (1989). CA 125 as an independent prognostic factor for survival in patients with epithelial ovarian cancer.. Am J Obstet Gynecol.

[OCR_00579] Zhou D. J., Ahuja H., Cline M. J. (1989). Proto-oncogene abnormalities in human breast cancer: c-ERBB-2 amplification does not correlate with recurrence of disease.. Oncogene.

